# Stress and temperature dependence of the avalanche dynamics during creep deformation of metallic glasses

**DOI:** 10.1038/srep33503

**Published:** 2016-09-22

**Authors:** Carlos Herrero-Gómez, Konrad Samwer

**Affiliations:** 1 Physikalisches Institut, Georg-August Universität Göttingen, Friederich-Hund-Platz 1, 37077 Göttingen, Germany

## Abstract

The understanding of the mesoscopic origin of plasticity in metallic glasses remains still an open issue. At the microscopic level, Shear Transformation Zones (STZ), composed by dozens of atoms, have been identified as the basic unit of the deformation process. Macroscopically, metallic glasses perform either homogeneous or inhomogeneous flow depending on the experimental conditions. However, the emergence of macroscopic behavior resulting from STZ interactions is still an open issue and is of great interest. In the current work we present an approach to analyze the different interaction mechanisms of STZ’s by studying the statistics of the avalanches produced by a metallic glass during tensile creep deformation. We identified a crossover between different regimes of avalanches, and we analyzed the dependence of such crossover on the experimental conditions, namely stress and temperature. We interpret such crossover as a transition from 3D random STZ activity to localized 2D nano-shear bands. The experimental time at which the crossover takes place seems to depend on the overall strain and strain rate in the sample

Amorphous materials have been known and used by mankind since ancient times^1^, but several aspects of their physics remain nowadays elusive^2^. Among those, the mesoscopic origin of plasticity in disordered materials in general, and Metallic Glasses (MG) in particular, is still debated^3^. MG´s are one of the most promising amorphous materials for industry applications due to their outstanding mechanical^4^ magnetic^5^ and chemical properties^6^. They present extremely high elastic limit, but one of their main drawbacks is their brittle nature at room temperature^7^,^8^. Unraveling the dependence of such macroscopic properties on the microscopic level is not only an open scientific topic, but is also crucial to foster industrial applications of MGs.

According to Argon’s model, the unit of the deformation process in an amorphous solid is the so-called Shear Transformation Zone (STZ)^9^,^10^. Such STZ’s have been experimentally observed in colloidal glasses^11^. Through an STZ process, the stress is relaxed by the collective shear of an ensemble of around 100 atoms, producing a long ranged stress Eshelby field with quadrupolar symmetry^12^. For the production of a small shear band and his nucleation a cooperative activation of those Eshelby inclusions is needed^13^. Under uniaxial tensile conditions, such alignment should take place nearly at 45° along the highest stress component. Once the shear bands are formed, additional processes like cavity formation are important for the failure of the sample, via cracks^14^. Therefore, the cooperativity or interaction of the STZ’s during the deformation turns out to be a key factor for the macroscopic behavior of the material.

In recent years, the plasticity of solid materials has been extensively studied in the framework of avalanche dynamics^15^,^16^,^17^,^18^,^19^. Stress-drops in the plastic regime of stress-strain curves of MG’s rods have been attributed to tuned criticality^20^. Shear avalanches have also been reported in nano-indentation experiments^21^. Furthermore, there have been several attempts to study the units of the deformation process through different theoretical approaches^22^,^23^,^24^. Apart from that, avalanche dynamics have been observed and studied in very different systems or situations, such as earthquakes^25^, neuronal networks^26^ or even some economy models^27^. In all cases, avalanche dynamics are characterized by the jerky response of a system subjected to a continuous or constant excitation^28^. This ragged response takes place without a characteristic scale, which is manifested in a power-law distribution of events sizes and durations. The exponents of those power-law distributions are believed to be universal and to depend on the type and dimensionality of the interactions present in the system^29^.

In a previous work, a study of the avalanche dynamics in a metallic glass upon a creep experiment was reported^30^. The main result was the finding of a crossover between two regimes of power-law scaling of the waiting time distribution. The waiting time was defined as the time required by the sample to deform 15 nm, which is the resolution limit of the device. That crossover took place between the exponents *τ* = −1.5 and *τ* = −0.8. Such crossover was interpreted as a change in the underlying mechanism of STZ’s interaction. According to that interpretation, during the first regime the STZ’s interact via stress-assisted randomly distributed avalanches (3D), and the second regime was attributed to an exhaustion of the first mechanism and its substitution by thermally activated (2D) correlated events.

The aim of the present work is to analyze the effect of the stress and temperature on the avalanche dynamics and the crossover between the two aforementioned regimes based on the waiting time analysis described in ref. 30 and to discuss the results based in the Potential Energy Landscape model (PEL)^31^,^32^,^33^. According to that picture, the stress decreases the energy barriers of the PEL and enhances the thermal activation of STZ’s which might assist a cooperative process^10^.

Here, we performed a set of creep experiments in *Pd*_77.5_*Cu*_6_*Si*_16.5_ MG samples for a broad set of values of stress and temperature. In each of the experiments a freshly prepared 40 *μm* thick ribbon was subjected to a constant load and temperature during a week in order to provide reliable statistics. We find a significant variation of the crossover time with increasing temperature and stress. After the mechanical testing we made sure by X-ray measurements that the samples were not crystallized nor oxidized.

## Experimental set-up

**Figure 1:**
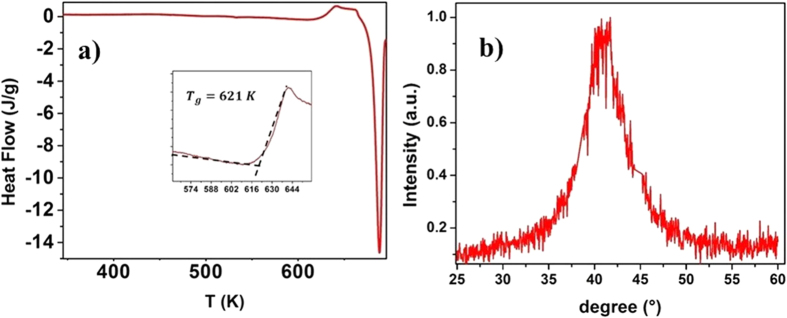
(**a**) DSC scan of a Pd_77.5_Cu_6_Si_16.5_ Metallic glass sample. (**b**) and X-ray Difraction pattern of a Pd_77.5_Cu_6_Si_16.5_ sample.

40 *μm* thick *Pd*_77.5_*Cu*_6_*Si*_16.5_ ribbons were prepared by arc-melting and melt spinning, their amorphous structure was checked by X-Ray Diffraction using a D8000 XRD from Siemens and it glass temperature was measured by DSC as is depicted in Fig. 1.

The creep experiments were performed in a Dynamic Mechanical Analyzer (DMA-7) by Perkin Elmer, placed in a glove box in *N*_2_ atmosphere to avoid oxidation at high temperatures. In those experiments the deformation as a function of time for a constant tensile stress and temperature was measured. Since the DMA lacks the resolution to resolve single microscopic elongation events (the spatial resolution is 15 nm), we analyzed the waiting times, which are defined as the elapsed time between two consecutive resolvable elongation jumps. The machine noise was filtered out using the same algorithm as described in ref. 30.

## Experimental Results

**Figure 2:**
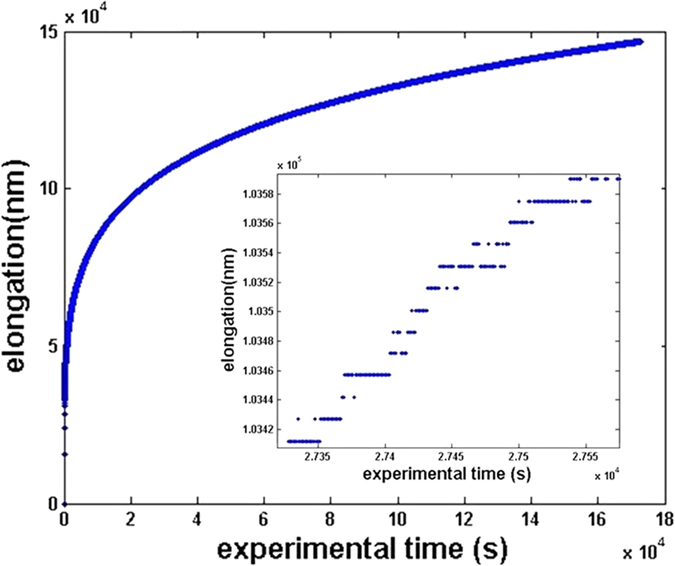
Creep measurement at T = 320 °C and σ = 20 MPa and its fine structure (inset).

Figure 2 shows a typical creep curve measured at T = 593 K and σ = 20 MPa. Although the elongation-time curve seems non linear but homogeneous on a global view, a closer look into the fine structure of the data shows a ragged and jerky shape, revealing a non-periodical distribution of jumps, which can be interpreted as a distribution of high and low strain rates.

**Figure 3:**
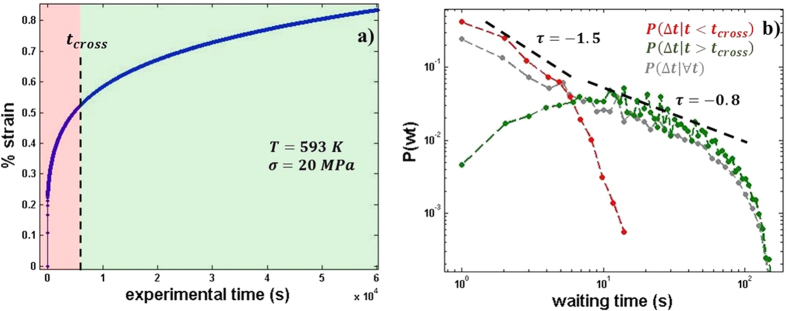
(**a**) Illustration of the crossover in the creep curve. (**b**) Waiting time distribution before the crossover: P_1_(*Δ*t|t < t_cross_), after the crossover: P_2_(*Δ*t|t > t_cross_) and for the whole experimental time 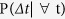.

A closer look to the waiting time distribution shows a crossover, where the scaling evolves with time from a power law with *τ* ~ −1.5 in the early stages of the experiment to *τ* ~ −0.8 later on (see Fig. 3b). Therefore, *t*_*cross*_ can be defined as an estimation of the experimental time in which the scaling of *P*(*Δt*) crosses over from *P*(*Δt*) ∝ *Δt*^−1.5^ to *P*(*Δt*) ∝ *Δt*^−0.8^. We defined *t*_*cross*_ as the time that divides the data in two subsets (*t* < *t*_*cross*_) and (*t* > *t*_*cross*_) in such a way that minimizes the error of the fit of the experimental waiting time distributions of both subsets *P*_1_(*Δt*|*t* < *t*_*cross*_) and *P*_2_(*Δt*|*t* > *t*_*cross*_) and a perfect power law distribution 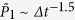 and 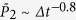 respectively. Figure 3a describes the crossover for the experiment corresponding to T = 593 K and σ = 20 MPa in the classical creep curve. Figure 3b shows the waiting time distributions *P*_1_(*Δt*|*t* < *t*_*cross*_) and *P*_2_(*Δt*|*t* < *t*_*cross*_) corresponding to the subsets of the data corresponding to before and after the crossover, together with the total distribution of waiting times. In Fig. 3a no clear signature of crossover can be seen, but Fig. 3b illustrates that the crossover is a necessary condition to describe the data set in power laws of *P*(*Δt*) ∝ *Δt*^−*τ*^.

**Figure 4:**
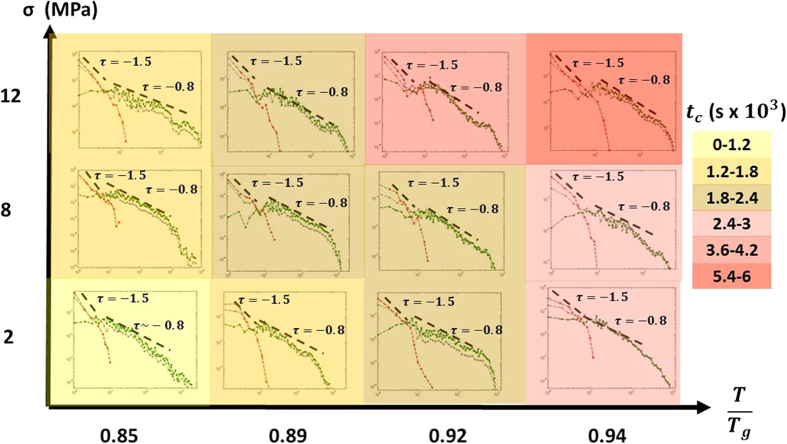
Stress-temperature map. Each square corresponds to a creep measurement under the corresponding 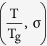 conditions. The background color represent the value of t_cross_.

To check if the crossover take places under different experimental conditions, we performed a set of experiments for different values of stress and temperature in the range *σ* = {2–12} and 
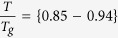
. In each of the experiments a freshly prepared sample for the very same sample batch was subjected to a constant stress and temperature and the crossover time was estimated in the aforementioned described way. Figure 4 shows the stress-temperature map. Each square corresponds to a different creep measurement in which *P*_1_(*Δt*|*t < t*_*cross*_), *P*_2_(*Δt*|*t* *>* *t*_*cross*_) and the distribution of the whole set of waiting times is displayed. Finally, the background color represents the *t*_*cross*_ value. The power laws always start with a *τ* ~ −1.5 dependence and shows later on a *τ* ~ −0.8 dependency. Therefore *t*_*cross*_ is the estimation of the experimental time in which the *τ* ~ −0.8 distribution is observed. It can be seen how *t*_*cross*_ increases both with temperature and stress.

**Figure 5:**
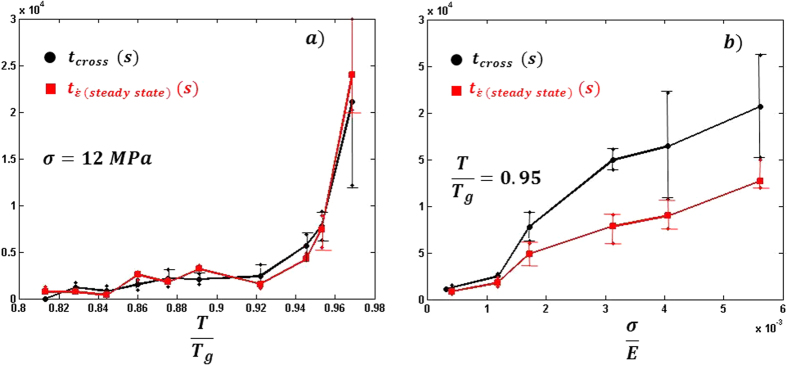
(**a**) Dependence of t_cross_ (black dots) and 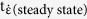 (red circles) with the temperature at a fixed stress. (**b**) Dependence of t_cross_ (black dots) and 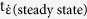 (red circles) with the stress at a fixed temperature. The errors bars indicate the range in which the fitted exponent take the values τ = 1.5 ± 0.1.

Finally in order to analyze in detail the dependence of *t*_*cross*_ with the stress and temperature, we explored further the σ-T space by adding two sets of experiments to the results of Fig. 4. In the first one, the stress was kept constant (σ = 12 MPa) among the different experiments and the temperature was swept through the range 
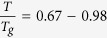
. In the second run, the temperature is kept constant 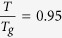 and instead, the stress was swept in the range σ = 2–35 MPa, which corresponds to 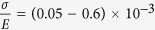, if we normalize by the Young modulus at that temperature. Figure 5 shows the dependence of *t*_*cross*_ with the stress and temperature.

Additionally, it can be observed in Fig. 3 that although the strain vs time curve and it derivative (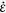 (t) vs t) are continuous functions, the average strain rate 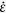 (t) decreases dramatically with time at early experimental times, and eventually it reaches a steady state value. From the derivative of the macroscopic creep curves from the aforementioned experiments, we calculated the strain rate as a function of time 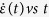. We observed a decrease of the overall strain rate of one order of magnitude 
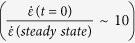
 for all our experiments. We also estimated from the 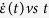 curves the experimental time at which the sample reaches such steady state of lower strain rate 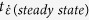. Both quantities *t*_*cross*_ and 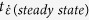 show a clear correlation as can be seen in Fig. 5 in which both are displayed.

Figure 5a shows that at 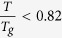 no crossover can be seen within our experimental resolution. Therefore, if there is any crossover it must be faster than 1 × 10^3^ *s*, but we are not able to observe any transition with good statistics. It also can be seen that as the temperature approaches the glass transition, *t*_*cross*_ increases rapidly. On the other hand Fig. 5b shows how *t*_*cross*_ increases in a more uniform fashion with the applied stress. When Fig. 5a is plotted in an Arrhenius-like fashion no clear 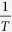 dependence can be seen and the activation energy would be *E*_*A*_ ~ 0.8 eV, close to the one corresponding to diffusion jumps and suggests that the crossover is a combination of a thermal and mechanical process. Finally the clear correlation between *t*_*cross*_ and 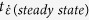 suggests a dependence of the crossover with the overall strain rate.

## Discussion

In ref. 30 the existence of a crossover from two different scaling regimes of the waiting times during a creep experiment was reported. It was argued that such crossover can be related to a change on the microscopic deformation mechanism in the MG. Such transition would be in agreement of the picture of a crossover from an uncorrelated 3-dimensional stress-driven plastic events regime (that would correspond to the *τ*_1_ ~ −1.5 regime) to a regime in which the main deformation mechanism is the thermal assisted collective nano-shear band activity in 2-dimensions (*τ*_2_ ~ −0.8).

Figure 4 points out that such crossover can be found under several experimental conditions. According to the model proposed in ref. 30 that means that in all that cases the deformation mechanism of the sample changes with time. It would start by a random activation of STZ’s in the space, and once the STZ density reaches a critical value, the STZ’s would cooperate forming 2-dimensional nano-shear bands.

The dependence of *t*_*cross*_ with temperature and stress is depicted in Fig. 5. Figure 5a shows how *t*_*cross*_ remains roughly constant or changes only slightly for low temperatures, and it increases dramatically as the temperature is increased approaching *T*_*g*_. On the other hand, the data displayed in Fig. 5b show that *t*_*cross*_ increases in a roughly linear fashion with the applied stress. In both cases there is a clear correlation between the crossover time *t*_*cross*_ and the time at which the overall strain rate saturates to a constant value 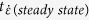.

It is known that macroscopic shear banding events are strongly affected by the strain rates. Those events tend to be intermittent at lower strain rates and successive at higher ones, leading to more pronounced serrations at low strain rates^34^.

Such strain rate dependence supports the interpretation of a transition from 3D STZ’s to 2D nano shear bands for the crossover in the creep experiments. The good correlation of *t*_*cross*_ with 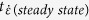 suggests that the system requires a constant strain rate to perform 2D nano shear bands.

The results depicted in Fig. 5 can also be interpreted in terms of the Eshelby correlations of the STZ’s. A decrease of the correlations strength at temperatures approaching *T*_*g*_ is expected due it randomizing effect and the acceleration of the relaxations. Such an effect is in agreement with the results shown in Fig. 5a. On the other hand the delay of the crossover at high stresses can also be addressed by the effect of the higher overall strain in the sample. Such increase of strain according to the uniaxial stress applied to the sample follows the stress under approximately 45° inside the sample. Therefore, it reduces the interaction among STZ’s via their Eshelby´s stress field, which decreases with a 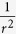
dependence. That implies that the onset of cooperativity among the STZ’s could be significantly delayed with a linear stress dependence and therefore the crossover might take place at a later time as the stress is increased while keeping the temperature constant.

## Methods

### Sample preparation

The glassy *Pd*_77.5_*Cu*_6_*Si*_16.5_ samples were prepared using arc-melting and melt-spinning method. The thickness of the samples are roughly 40 μm. The glass transition was measured by DSC and was found at 621 K.

### DMA creep measurements

The experiments were perfomed on samples mounted in metallic holders and clamped in a PerkinElmer DMA 7.A minimum pre-estress of 1.5 MPa was applied to keep the samples straight and stable during the heating process. The creep experiment was not started until the temperature reached a stable value. Once the temperature was stable a constant stress was applied and the elongation as a function of time was measured. The DMA7 was placed in a glove box in *N*_2_ atmosphere to avoid oxidation of the samples.

### Waiting time analysis and crossover

The machine noise was filtered using the same procedure as proposed in ref. 30. The error bars of the estimated crossover time in Fig. 5 correspond to the interval of time in which *τ*_1_ = 1.5 ± 0.1.

## Additional Information

**How to cite this article**: Herrero-Gómez, C. and Samwer, K. Stress and temperature dependence of the avalanche dynamics during creep deformation of metallic glasses. *Sci. Rep.* **6**, 33503; doi: 10.1038/srep33503 (2016).
